# Rat Hepatitis E Virus: Presence in Humans in South-Western France?

**DOI:** 10.3389/fmed.2021.726363

**Published:** 2021-09-03

**Authors:** Delphine Parraud, Sébastien Lhomme, Jean Marie Péron, Isabelle Da Silva, Suzanne Tavitian, Nassim Kamar, Jacques Izopet, Florence Abravanel

**Affiliations:** ^1^Virology Laboratory, National Reference Centre of Hepatitis E Viruses, Federal Institute of Biology, University Hospital, Toulouse, France; ^2^Institut Toulousain des Maladies Infectieuses et Inflammatoires (Infinity) INSERM UMR1291–CNRS UMR5051–Université Toulouse III, Toulouse, France; ^3^Department of Gastroenterology, Rangueil University Hospital, Toulouse, France; ^4^Department of Hematology, Cancer University Institute of Toulouse, Toulouse, France; ^5^Departments of Nephrology and Organ Transplantation, Rangueil University Hospital, INSERM U1043, IFR-BMT, University Paul Sabatier, Toulouse, France

**Keywords:** hepatitis E virus, rat hepatitis E virus, immunocompromised, South-Western France, chronic hepatitis, hepatitis E, zoonosis, rodent

## Abstract

**Background:** Hepatitis E Virus (HEV) is one of the most common causes of hepatitis worldwide, and South-Western France is a high HEV seroprevalence area. While most cases of HEV infection are associated with the species Orthohepevirus-A, several studies have reported a few cases of HEV infections due to Orthohepevirus-C (HEV-C) that usually infects rats. Most of these human cases have occurred in immunocompromised patients. We have screened for the presence of HEV-C in our region.

**Methods and Results:** We tested 224 sera, mostly from immunocompromised patients, for HEV-C RNA using an in-house real time RT-PCR. Liver function tests gave elevated results in 63% of patients: mean ALT was 159 IU/L (normal < 40 IU/L). Anti-HEV IgG (49%) and anti-HEV IgM (9.4%) were frequently present but none of the samples tested positive for HEV-C RNA.

**Conclusion:** HEV-C does not circulate in the human population of South-Western France, despite the high seroprevalence of anti-HEV IgG.

## Introduction

Hepatitis E virus (HEV) is a major cause of epidemic waterborne and sporadic hepatitis worldwide (1). HEVs belong to the family Hepeviridae, which contains two genera: Orthohepevirus and Piscihepevirus (1). Genus Orthohepevirus comprises four species: Orthohepevirus A–D. The viruses that make up the species Orthohepevirus A are assigned to one of at least eight distinct genotypes (HEV-1 to HEV-8); the four major ones are the main causes of infection in human (1).

Hepatitis E produces a benign infection in most immunocompetent people, with a wide range of symptoms, including fever, nausea, anorexia, asthenia, vomiting, abdominal pain, and icterus. Liver function tests are often abnormal, with signs of hepatic cytolysis (increased transaminases), cholestasis, and sometimes even liver failure. Severe acute or fulminant hepatitis is rare, usually occurring in patients with underlying chronic liver disease and pregnant women with an HEV-1 or HEV-2 infection (2). The virus can also produce extra-hepatic symptoms like neurological disorders, renal failure, pancreatitis, and hematological disorders (1). HEV replication can persist for over 3–6 months in adults or children on immunosuppressive therapy following transplantation, those on chemotherapy or immunotherapy, and persons with a concomitant HIV infection. Such infections can lead to chronic hepatitis with progressive liver fibrosis and cirrhosis. The majority of immunosuppressed patients are asymptomatic and present with persistent, mildly abnormal liver function tests (1).

In developed countries, HEV is a zoonosis with pigs being the main reservoir (1). Direct and indirect contact with infected animals or consumption of contaminated food products are the main transmission routes of HEV. Rabbit HEV, which is closely related to HEV3, circulates in rabbits in China, the United States and Europe (3, 4). Other food products are also possible sources of HEV, as HEV3 RNA was detected in red fruits, strawberries, green leaves and spices (1). Furthermore, one case of transmission of HEV7 to an immunocompromised individual through consumption of camel meat and milk has been described (5). HEV RNA was recently identified in goat and sheep milk and could represent a source of infection to consumers (6). Parenteral transmission by blood transfusion is also a potential mode of contamination (1).

An HEV of rat origin has recently been shown to cause zoonotic infections and symptomatic disease (hepatitis) in humans (7). This rat HEV is a genetically distant relative of other mammalian HEVs; it is about 55–60% sequence homologous to HEV genotypes 1–4, and is assigned to the genus Orthohepevirus C (7). In Europe, studies conducted on Norway rats and Black rats, reported the detection of HEV-C RNA with prevalence ranging between 0.3 and 27.2% (8–15); HEV-C RNA was also frequently detected in Asia with prevalence ranging between 0.7 and 26.3% in rats (16–21), 1.9% in musk shrew (22) and between 1.5 and 7.8% in 2 US studies in rats (23, 24). The virus isolated in musk shrew could infect nude mice (25). The first human case identified was a previously healthy Canadian man (26) and there have been a few recent (2017–2020) confirmed cases in Hong Kong (27–29). In the largest epidemiological studies performed in Honk-Hong, HEV-C1 RNA was detected in 6/2,201 (0.27%) patients with hepatitis and 1/659 (0.15%) immunocompromised persons (28). The eight HEV-C1 infections identified included 3 with acute hepatitis, four with persistent hepatitis, and one with a subclinical infection but no hepatitis. Significantly, seven of the eight patients were immunosuppressed (28). The source of transmission from rat to these human cases is still unknown. None of these patients had a history of rat meat consumption, and the practice is uncommon in Hong Kong. Indeed, almost all of them even denied rat infestation in their domestic premises. The adulteration of food products or natural HEV-C infection of pigs may be a possibility (28). Another relevant sanitary aspect of rat as HEV carrier is the possibility of interspecies transmission of HEV-3 between pigs, wild boar, humans and rats. Three studies reported the detection of HEV-3 in rats, in the USA, Japan and Belgium (10, 23, 30). Rats are synanthropic mammals and their ubiquitous presence in urban environments and in rural areas where pigs are farmed may explain the interspecies transmission.

As the seroprevalence of anti-HEV IgG is high among blood donors in our area (52.5%) (31), and HEV-A infections are frequent among immunocompromised patients (32), while cirrhotic patients are at high risk of hepatic decompensation (33) these populations in our area are regularly tested for HEV-A. This prompted us to assess the risk of an HEV-C infection.

## Patients and Method

### Patients Samples

We investigated prospectively 224 patients who tested negative for HEV-A RNA. They were from a group of individuals at risk of developing a chronic HEV infection, for whom physicians had prescribed regular HEV-A RNA tests. The majority of them were immunocompromised (organ transplant recipients, hematological malignancies, bone marrow transplant recipients and solid cancer). All sera were collected between December 2019 and May 2020 and all tested negative for HEV-A RNA. According to French law (Loi Jardé), because this is an anonymous study without additional blood sampling, institutional review board approval was not required.

### Serological Assays

We used commercial ELISA kits (Wantai, Beijing, China) for the serological tests for HEV-IgM and HEV-IgG as recommended by the manufacturer. The capture antigen in these kits is an ORF2 fragment of HEV-A genotype 1. We used the Wantai kits since they cross-react with both anti-HEV-A and anti-HEV-C antibodies (34). Sample were considered as positive if the signal to cut-off ratio (S/Co) was >1.

### HEV-C RNA Detection

RNA was extracted using QIAamp Viral RNA Mini kit (Qiagen, Courtabeuf, France). We diagnosed HEV-C infections using an in-house qRT-PCR assay based on HEV-C sequences available in Genbank.

Reverse transcription and amplification were performed using the SuperScriptIII Platinum One-Step qRT-PCR kit (Invitrogen). The forward primer was 5′-CCACGGGGTTAATACTGC-3′, the reverse primer was 5′-CGGATGCGACCAAGAAACAG-3' and the probe was 5'-6FAM-CGGCTACCGCCTTTGCTAATGC-BBQ-3'. Each 50 μL mix contained: RNA (15 μL), SuperScript III RT/Platinum TaqMix (1 μL), buffer containing 0.4 mM of each dNTP and 6 mM MgSO4, 0.4 μM forward and reverse primers, 0.2 μM probe, and 1 μL RNAse-out. The RT-PCR cycle was: 50°C/25, 95°C/10 min, 95°C/20, and 56°C/45 s. HEV RNA was detected on a Light Cycler 480 Real-Time PCR System (Roche).

The rat HEV-RNA positive control was extracted from a PLC/PRF/5 cell culture supernatant containing rat HEV strain 63 (kindly provided by Prof. Reimar Johne).

## Results

The 224 samples analyzed included 207 from immunocompromised patients. Of these, 67 were solid organ recipients, 48 suffered from hematological malignancies, 85 were bone marrow transplant recipients, 5 had solid cancers, 2 were on corticosteroids and 17 were immunocompetent with abnormal liver function tests (including 4 cirrhotic patients). Over half the patients (142/224; 63%) had elevated liver function tests (mean ALT: 159 IU/L) (Table 1).

**Table 1:** Clinical and demographic features of patients.

Mean age (year)	53 ± 17
Gender (Male/Female)	133/91
**Underlying pathologies**	
Bone marrow transplant recipients	85
Organ transplant recipients	67
Hematological malignancies	48
Solid cancer	5
Cirrhosis	4
Corticosteroids treatment	2
Others	13
ALT (IU/L)	159 ± 378
AST (IU/L)	162 ± 753
Bilirubine (μmol/L)	239 ± 45
GGT (IU/L)	20 ± 488
IgG VHE+	110/224
IgM VHE +	21/224
Total (*n*)	224

**Figure 1:**
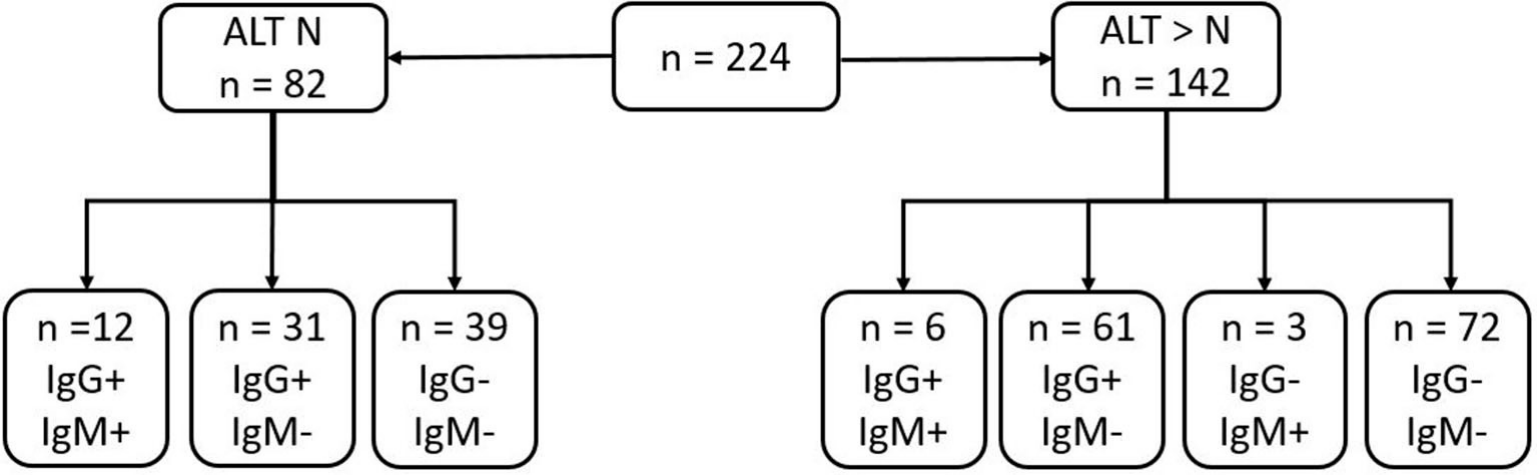
Summary of serological results of samples depending on ALT results (N, normal value: *N* < 35 U/L in our lab).

**Figure 2:**
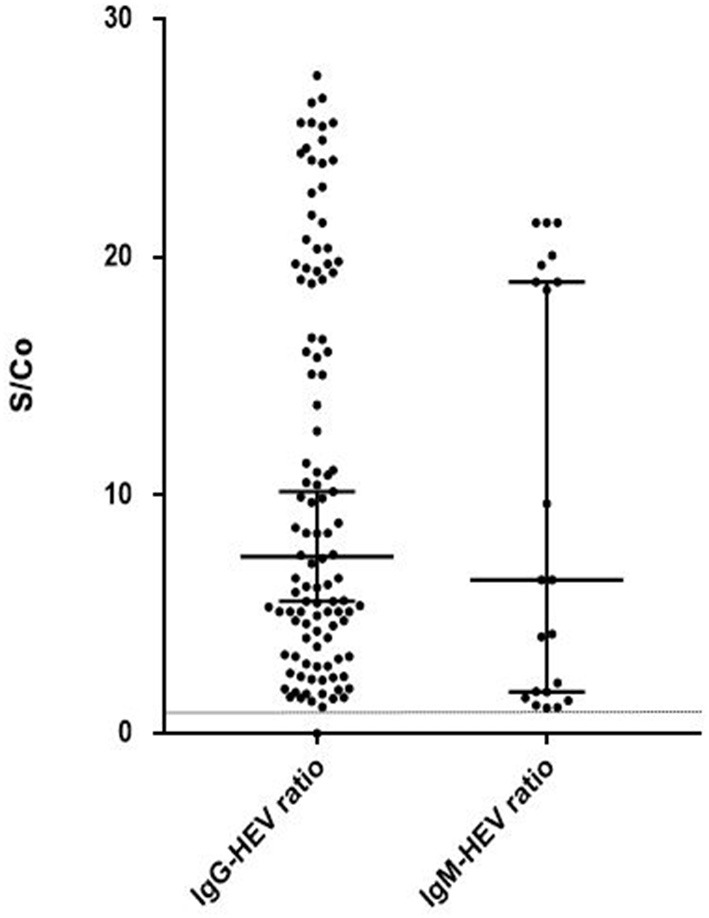
HEV IgG and IgM ratio (S/Co) in positive samples (S/Co:signal-to-cut-off ratio). Limit of positivity is plotted on the vertical axis with the dotted line.

Summary of serological results are presented in Figure 1. Anti-HEV IgG was present in 49% of the sera and anti-HEV IgM in 9.4%. The ratios of the HEV IgG and IgM are presented in Figure 2. The sera from 14.6 % (12/52) of the patients with normal liver function tests contained anti-HEV IgM, as did the sera from 6.3% (9/142) of those with an elevated ALT.

All samples tested negative for HEV-C RNA.

## Discussion

Our investigation of individuals, mainly immunosuppressed, who were highly susceptible to viral infections found no HEV-C infections, even in the patients with anti-HEV antibodies or transaminase elevation.

Most of the patients we tested were immunosuppressed and had been referred by their physician for an HEV diagnosis as this population is at risk of persistent HEV-A infection or HEV-C1 infection, as recently reported by Sridhar et al. (28). They found that among the 8 cases of HEV-C infection included 7 were immunocompromised and 3 of them developed a chronic infection. Moreover, prior exposure to HEV-A does not protect against an HEV-C1 infection (34).

Sridhar et al. recently investigated the divergence between HEV-C1 and HEV-A, the usual cause of human hepatitis (34) because genomic divergence reduces the capacity of existing tests to diagnose HEV-C. Human HEV-based antigen and antibody assays, even molecular assays, may not diagnose HEV-C1 infections. We therefore developed an in-house RT-PCR assay to look for HEV-C RNA and tested the samples for anti-HEV-C antibodies using the Wantai IgM and IgG kits, which cross-react with both anti-HEV-A and anti-HEV-C antibodies (34).

There were 21 HEV-IgM positive patients, none of whom had any virus detected, either HEV-A or HEV-C, by PCR. They probably had a HEV-A infection in the past, as HEV-IgM is a persistent marker that can be detected up to 3 years after acute hepatitis (35). Similarly, HEV-IgG can persist for a long period after an HEV-A infection, up to 14 years (1). Additionally, 142/224 (63.4%) of our patients had abnormal aminotransferase activities, but they tested negative for HEV-RNA. Only one of them tested positive for HCV RNA. Our patients may have several causes of transaminases elevation: drug induced liver injury, graft-vs. diseases cirrhosis.

Despite the high HEV-A seroprevalence in South-Western France, HEV-C does not seem to circulate in the human population. However, we focused our study in immunocompromised patients and other groups of patients should be investigated. The epidemiology of HEV-A infections is known to vary greatly from one geographic area to another. The route of transmission between rats and humans in Hong Kong, where the majority of the HEV-C infections have been reported, is still elusive. Certain populations are known to be more at risk of infection, depending on immunocompromised factors and/or life style. One study found a few forestry workers in Germany tested positive for anti-HEV-C antibodies (36). Thus, serological and molecular assays that discriminate between HEV-A and HEV-C would be valuable for determining the real distribution of rat HEV worldwide. Immunoblot assays could help to distinguish the serological profiles of HEV infections (34).

Sridhar et al. reported that HEV-C infections accounted for 8% of all genotyped hepatitis E cases in Hong Kong (28, 34). We therefore expected to find at least some cases of HEV-C1 among the limited number of patients tested, as anti-HEV antibodies are prevalent in our region, particularly in the population investigated. We have designed our RT-PCR assay to detect all HEV-C variants according to the sequences available in Genbank. We used a culture supernatant of a rat HEV to verify the ability of our test to detect this specific species, but we cannot rule out the possibility that our PCR have missed a divergent strain.

In conclusion, we found no case of rat HEV infection between December 2019 and May 2020 despite the high seroprevalence of HEV in our region. A larger study, testing patients with occupational exposure and more immunocompromised patients, using discriminatory serological assays such as immunoblots, could help reveal the presence of HEV-C in South-Western France. Specific molecular assays would also enable us to confirm human cases of HEV-C if serological tests were positive.

## Data Availability Statement

The raw data supporting the conclusions of this article will be made available by the authors, without undue reservation.

## Ethics Statement

Ethical review and approval was not required for the study on human participants in accordance with the local legislation and institutional requirements. Written informed consent for participation was not required for this study in accordance with the national legislation and the institutional requirements.

## Author Contributions

JI, DP, and FA: drafting and refining the manuscript. SL: critical reading of the manuscript. DP, FA, JP, ST, and NK collected the data. ID performed the analysis. All authors contributed to the article and approved the submitted version.

## Conflict of Interest

The authors declare that the research was conducted in the absence of any commercial or financial relationships that could be construed as a potential conflict of interest.

## Publisher's Note

All claims expressed in this article are solely those of the authors and do not necessarily represent those of their affiliated organizations, or those of the publisher, the editors and the reviewers. Any product that may be evaluated in this article, or claim that may be made by its manufacturer, is not guaranteed or endorsed by the publisher.

## Abbreviations

ALT: alanine aminotransferase
HEV: hepatitis E virus
RNA: ribonucleic acid.
